# Analysis of Lumbar Joint Movement and Dynamics of Weightlifters

**DOI:** 10.1155/abb/3678401

**Published:** 2025-10-21

**Authors:** Li Xiao

**Affiliations:** ^1^ Department of Basic Teaching, Henan Logistics Vocational College, Zhengzhou, 450000, China

**Keywords:** finite element analysis, hard pull actions, kinematics, lumbar joint, weightlifting

## Abstract

**Objective:**

To analyze the effects of different hard pull actions on lumbar joint movement and dynamics in weightlifting and evaluate their contribution to the risk of lumbar spine injury.

**Method:**

The study recruited eight national second level and above weightlifters as volunteers, and conducted detailed kinematic and dynamic analysis of different hard pull actions through exercise experiments and finite element analysis (FEA).

**Result:**

The lumbar flexion angles of traditional hard pull and hexagonal barbell hard pull were 58° and 55°, respectively, while the lumbar flexion angle of straight leg hard pull was 90°. The first peak torque of straight leg hard pull was 893 N∙m, significantly higher than the traditional hard pull of 749 N∙m and the hexagonal barbell hard pull of 640 N∙m. In terms of stress distribution, the peak stress of straight leg hard pull at the L5 vertebral body was 997 MPa, and the peak stress of L5 trabecular bone was 3.3 MPa, both higher than traditional hard pull and hexagonal barbell hard pull. The peak stress of the lumbar intervertebral disc was also highest in straight leg tension, with the peak stress of the L4–5 lumbar intervertebral disc being 45.6 MPa.

**Conclusion:**

Straight leg hard pull causes greater damage to the lumbar spine due to its larger lumbar flexion angle and higher peak stress. The damage to the L5 vertebral body during weightlifting is much higher than that of other lumbar vertebrae. Therefore, it is recommended to reduce straight leg hard pull during weightlifting training and strengthen protective measures for the L5 vertebral body to reduce lumbar spine injury.

## 1. Introduction

Weightlifting, as an ancient sport with a history dating back to ancient civilizations, is widely regarded as an effective way to enhance strength, muscle volume, and overall physical fitness. Since the first modern Olympic Games in 1896, weightlifting has been listed as an official competition event, and its position in sports competition is self‐evident [1, 2]. Over time, weightlifting has developed into a comprehensive strength training that includes both snatch and clean and jerk forms, not only occupying a place in competitive sports but also widely popular in the field of mass fitness. As a part of the spine, the lumbar spine exerts a crucial role in weightlifting. It affects the performance of weightlifting, and also relates to the health and safety of athletes [3, 4]. During weightlifting, strength is transmitted from the lower limbs to the upper limbs and barbell through the lumbar spine. However, due to the long‐term training and competition of weightlifters, the lumbar spine is subjected to enormous pressure and load, making it one of the most vulnerable areas to injury. These injuries affect athletes’ competitive performance and also have impacts on their long‐term health.

Aasa et al. [5] analyzed the variability of upper and lower lumbar spine alignment during hard lifts and squats. The results showed that the correlation coefficient range of dead lift was between 0.69 and 0.99, and the absolute reliability of the minimum detectable change was between 1° and 8°. When repeating three dead lifts and squats, the lumbar alignment of the weightlifter did not change much between repetitions [5]. Scott et al. [6] analyzed the impact of spinal warning information and spinal elasticity information on maximum dead lift performance. Compared to participants who received warning education, those who received resilience education had a threefold increase in their belief in spinal fragility. Brief flexible education may have a positive impact on beliefs about spinal fragility [6]. Sjöberg et al. [7] analyzed the lumbar spine load and muscle activity during flywheel and barbell leg movements. During flywheel action, the peak force of the lumbar multifidus muscle was lower than during barbell action. The peak electromyographic activity range was between 31% and 122% of the maximum autonomous isometric contraction under muscle and exercise modes, with the highest level observed in the lumbar multifidus [7]. Although existing research has focused on lumbar injuries in weightlifting, comparative studies on the specific effects of different hard pull actions on the lumbar spine are still limited.

Biomechanics, as a science that applies principles of physics and engineering to analyze the movement of organisms, mainly focuses on the forces, moments, energy, kinematics, and dynamic characteristics involved in the movement of organisms. It plays an important role in improving sports performance, preventing sports injuries, optimizing training methods, and improving sports techniques. Ke et al. [8] used finite element analysis (FEA) and contour visualization methods to analyze the biomechanical effects of small joint parameters on the corresponding segments of the lumbar spine. The results showed that when there was a small joint orientation, the stress on the intervertebral disc was concentrated in the ipsilateral area of the small joint with a larger sagittal direction. The orientation of small joints with higher sagittal planes may increase recurrent lumbar degenerative changes due to increased pressure on the ipsilateral intervertebral disc [8]. Spina et al. [9] took FEA to analyze the biomechanical effects of lumbar laminectomy. With the increase of bone resection, the stress in the joint area increased nonlinearly [9]. Zhao et al. [10] used FEA to analyze the biomechanical effects of different bagging methods on the lumbar spine and paraspinal muscles. Shoulder bags and handbags led to greater muscle strength, resulting in greater intervertebral compression force and intervertebral shear force in the lumbar spine [10].

In summary, although existing research focuses on the mechanism and prevention of lumbar spine injury in weightlifting, there are three key gaps: Firstly, most studies only focus on the lumbar spine load of a single hard pull movement, lacking a systematic comparison of the three mainstream training movements of traditional hard pull, straight leg hard pull, and hexagonal barbell hard pull, and unable to clarify the differences in lumbar spine injury risk between different movements; Secondly, although some studies involve biomechanical analysis of hard pulled lumbar vertebrae, they often rely on a single experimental method and have not achieved the combination of “high‐precision motion data acquisition + finite element simulation of segmented structures,” making it difficult to quantify the dynamic biomechanical response of lumbar vertebrae at various stages during the execution of movements. Therefore, to analyze the action characteristics of different hard pull actions, this study conducted biomechanical analysis of the lumbar spine using FEA method. The study recruited national second level and above weightlifters as volunteers, using advanced computer tomography equipment, 3D motion image acquisition systems, and 3D force measurement platforms, combined with software such as Solidworks, Mimics, Abaqus, etc., to conduct systematic kinematic and dynamic analysis of different hard pull actions. It is expected to reveal the effects of various hard pull actions on lumbar flexion angle, torque changes, and stress distribution, providing scientific basis for training methods, skill improvement, and injury prevention in weightlifting. The innovation of the research lies in: firstly, using three types of hard pulling as comparison objects, comprehensively revealing the risk gradient of lumbar spine injury for different movements, providing a direct basis for “action priority selection” in training. Secondly, refine the “action stage structural part” to achieve dual‐dimensional risk positioning.

## 2. Methods and Materials

### 2.1. Experimental Subjects and Methods

To analyze the kinematic characteristics of the lumbar spine of weightlifters, eight volunteers were recruited for exercise experiments. The recruitment criteria for volunteers are as follows: (1) volunteers must be at least Chinese national second level and above. (2) No history of lumbar spine injury within 6 months and in good health. All volunteers are Chinese national second level and above weightlifters, including 6 males and 2 females, aged 20–28 years (average age 24.3 ± 2.1 years), with a height of 165–182 cm (average 173.5 ± 5.8 cm), a weight of 68–85 kg (average 76.2 ± 6.3 kg), and a BMI index of 22.1–26.4 kg/m^2^ (average 24.2 ± 1.3 kg/m^2^). They all meet the physical characteristics of weightlifters and have no obvious physical development abnormalities or underlying diseases that affect motor function. At the same time, the study used actual weightlifting tests (nonsimulated weightlifting), and all hard pull movements were completed on standard weightlifting training grounds to ensure that the experimental scene was consistent with the athletes’ daily training environment. Table 1 displays the software and equipment required.

**Table 1:** Software and equipment required for the experiment.

Device/software name	Manufacturer	Configuration/parameters
Computer tomography equipment (Somatom Sensation 10)	Siemens AG	—
Computer workstation (XiaoXin Pro 16 ACH 2021)	Lenovo Group Co., Ltd	CPU: AMD Ryzen 7 5800H; physical memory: 16 GB; graphics card: NVIDIA GeForce MX450; operating system: Windows 10 64 bit
Solidworks 2019	Dassault Systemes	—
Mimics 21.0	Materialize	—
Abaqus 2021	Dassault Systemes	—
Geomagic Wrap 12.0	Geomagic	—
Visual 3D	C‐Motion Corporation, USA	—
Microsoft Excel 2016	Microsoft	—
SPSS 25.0	IBM SPSS	—
3D motion image acquisition system	Qualisys, Sweden	10 infrared cameras, sampling frequency: 100 Hz
3D Force measurement platform	KISTLER, Switzerland	Measurement plate size: 50 cm × 60 cm, acquisition frequency: 500 Hz
Metronome	NIKON CORPORATION	—
Barbell	China Biaohan Hardware Company	—
Tape measure	Deli Group Co., Ltd	—

According to Table 1, the required equipment for the experiment includes computer tomography equipment, computer workstation, 3D motion image acquisition system, 3D force measurement platform, metronome, and barbell. The required software includes Solidworks, Mimics, Abaqus, Geomagic Wrap, and Visual 3D. The test actions in the experiment include traditional hard pull action, straight leg hard pull action, and hexagonal barbell hard pull action.

The requirements for traditional hard pull actions: Firstly, the feet should be shoulder width or slightly narrower than shoulder width, and the toes should naturally point forward or slightly outward. Throughout the entire action, volunteers should keep their backs straight, avoid hunching or excessive bending, and their hips should be slightly lower than their shoulders. The barbell bar should be placed in the middle of the instep, and the grip method is to close and hold it upright. Before pulling up the barbell, the knees should be slightly bent, but not excessively bent. When exerting force, initiate the pulling action by pushing the heel and stretching the leg to keep the back straight [11, 12]. During the hard pulling process, avoid twisting the body and maintain the neutral position of the spine.

Requirements for straight leg hard pull action: Firstly, feet should be shoulder width or slightly narrower than shoulder width, and the toes should naturally point forward or slightly outward. Throughout the entire action, volunteers should keep their backs straight, avoid hunching or excessive bending, and their hips should be slightly lower than their shoulders. The placement and gripping method of the barbell are the same as traditional hard pulling. At the beginning of the action, the legs are almost straight and the knee joints are slightly bent to avoid joint locking [13, 14]. When exerting force, the pulling action should be initiated by pushing the heel and stretching the hips, rather than bending the knee joint.

The requirements for the hard pull action of the hexagonal barbell: Firstly, the standing position should be at the center back of the hexagonal barbell. The action posture should be consistent with traditional hard pull. When exerting force, the pulling action should be initiated by pushing the heel and stretching the hips, rather than bending the knee joint [15, 16].

In the experiment, the barbell is 120 kg, and the load weight is set to 75% of the maximum weight of a single traditional hard pull for volunteers. At the same time, for the convenience of subsequent analysis, the action is divided into four phases based on the changes in lumbar torque. First hard pull phase: from the beginning of hard pulling to the moment reaching its peak for the first time. Transition phase: from the end of the first peak to the first trough. Second hard pull phase: from the end of the first trough to the second peak. Standing phase: from the end of the second wave peak to fully standing upright.

The experimental steps are as follows: Firstly, volunteers need to warm up for 10 min before the test to ensure their physical fitness. Before testing, it is necessary to calibrate the spatial coordinate system, setting the sagittal axis (facing direction is positive) and coronal axis (left arm side is positive) of the human body as the X and Y directions, respectively. The Z‐axis (head direction is positive) is perpendicular to the X and Y directions. Afterwards, the force measuring platform is zeroed to ensure accuracy. At the same time, 49 reflective Mark points are pasted onto the subjects and the midpoint of the barbell for the infrared camera to capture the action trajectory. Table 2 displays the Mark point pasting position.

**Table 2:** Mark point paste locations.

Region	Position
Head	Glabella
	Above the left ear
	Above the right ear

Torso	Upper end of sternum stalk
	Upper sternum
	Seventh cervical vertebra
	Second thoracic vertebra
	Midpoint of scapular angle
	First lumbar vertebra
	Third lumbar vertebra
	5th lumbar vertebra

Pelvis (left and right)	Anterior superior iliac spine
	Posterior superior iliac spine

Barbell	Left midpoint
	Right midpoint

Upper limbs (left and right)	Shoulder peak end
	Middle segment of humerus
	Elbow joint
	Medial wrist joint
	Lateral wrist joint
	First joint of index finger

Lower limbs (left and right)	Greater trochanter of femur
	Medial and lateral condyles of femur
	Fibular head
	Medial condyle of tibia
	Medial condyle of ankle joint
	Lateral condyle of ankle joint
	Root Bone
	First mid phalangeal bone
	Third mid phalangeal bone
	5th mid phalangeal bone

According to Table 2, the Mark point pasting positions include the head, torso, upper limbs, pelvis, lower limbs, and barbell. After pasting the Mark points, the metronome is adjusted to swing at a frequency of 2 s/time. At the beginning of the testing phase, the data that needs to be collected for the hard pull action is first collected using a 3D motion image acquisition system with a 3D force measuring platform. When collecting motion data, volunteers need to maintain uniform clothing [17, 18]. After completing the initial static motion, the subject begins to perform a hard pull action until their body is fully extended and standing steadily, completing one hard pull action. During the entire testing process, the subjects will repeat the hard pull action multiple times, with a 2‐min interval between each time, to ensure safety and effectiveness, as well as the data accuracy and reliability. Eight volunteers participated in three types of hard pull tests, and the “Latin square random method” was used to determine the order of action testing (such as Volunteer 1: traditional hard pull → straight leg hard pull → hexagonal hard pull; Volunteer 2: straight leg hard pull → hexagonal hard pull → traditional hard pull), in order to avoid the “action sequence effect” (such as fatigue caused by high difficulty movements first). The interval between each movement test is 48 h to ensure the muscle recovery of volunteers (refer to the standard for weightlifting training recovery cycle) and to eliminate the interference of fatigue accumulation on lumbar biomechanical indicators.

### 2.2. Construction Method of FEA Model

**Figure 1:**
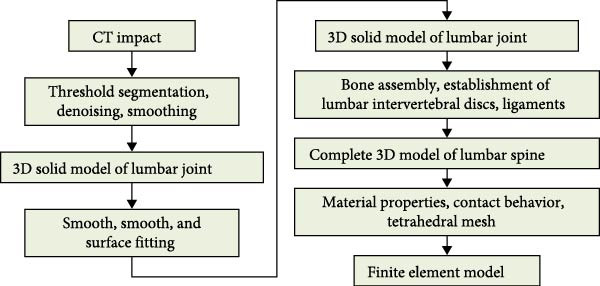
Construction process of FEA model.

To analyze the kinematic characteristics of the lumbar spine of weightlifters, a FEA of the lumbar spine is constructed using a volunteer as the object. Before constructing the FEA model, the imaging data of the lumbar spine is collected. The data collection method is as follows. Computerized tomographic (CT) scanning equipment is used to scan the volunteer’s torso from top to bottom, with a scan layer thickness of 0.63 mm. The specific method for collecting lumbar spine data is as follows: Siemens Somatom Sensation 10 spiral CT equipment is used for lumbar spine tomography. The scanning parameters are strictly controlled as follows: tube voltage of 120 kV, tube current of 250 mA, layer thickness of 0.63 mm, layer spacing of 0.3 mm, matrix of 512 × 512, field of view (FOV) of 350 × 350 mm, ensuring a spatial resolution of 0.68 mm/pixel and clear display of fine structures such as lumbar cortical bone, cancellous bone, and intervertebral discs. The scanning process is as follows: (1) The subject is placed in a supine position, with legs naturally extended and the waist positioned at the center of the scanning bed. A positioning laser line is used to calibrate the longitudinal axis of the lumbar spine to be parallel to the longitudinal axis of the scanning bed, avoiding image distortion caused by tilted positions; (2) The scanning range extends from the upper edge of the T12 vertebral body to the lower edge of the S1 vertebral body, covering the entire lumbar spine (L1–L5) and adjacent segments to ensure that there are no structural defects in subsequent model construction; After the scanning is completed, the images are stored in DICOM 3.0 format, and 1025–1050 tomographic images are generated for each subject. The file size of each individual image is ≥5 MB to ensure data integrity. 1025 images can be obtained through CT scanning and stored in DICOM format. Then, the collected CT images are imported into Mimics 21.0 to extract Hounsfield Unit (HU) values of bones and determine the spatial position of the images in the human body based on anatomical knowledge. After determining the spatial position of the planar image, threshold segmentation can be performed on the image. Figure 1 displays the FEA model construction process.

**Figure 2:**
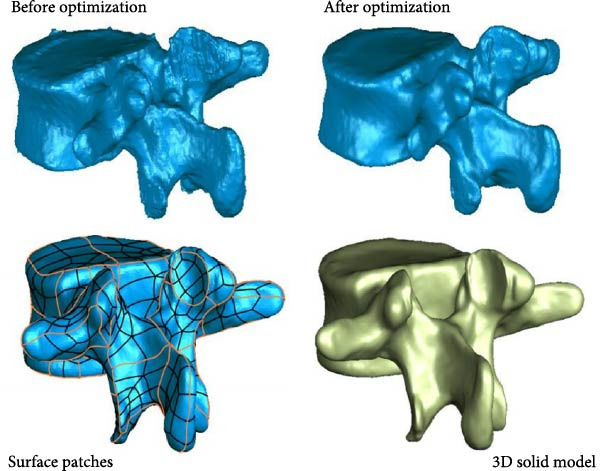
3D FEA model of L2 vertebrae.

In Figure 1, firstly, Mimics 21.0 software is used to perform threshold segmentation, denoising, and smoothing on the CT images of the lumbar spine joints of a male subject, to construct a 3D solid model of the lumbar spine joints. Then, Geomagic Wrap 12.0 software is taken to smooth and fit NURBS surfaces on the 3D solid model, generating a 3D geometric model of the lumbar spine joint. Next, Solidworks 2019 software is used for bone assembly, establishing lumbar intervertebral discs and ligaments, and constructing a complete 3D geometric model of lumbar joints. Finally, Abaqus 2021 software assigns material properties to the model, defines contact behavior, and establishes a tetrahedral mesh to obtain the FEA model of the lumbar spine. Due to the quality differences in CT images, there may be mixed vertebral tissue in the threshold segmentation results [19, 20]. Therefore, manual inspection is still required after threshold segmentation, and incomplete parts need to be filled in. By following the above steps, the initial 3D FEA model can be obtained. After denoising, smoothing, and optimizing the initial 3D finite element model, a 3D solid model can be obtained. The model is stored in STL format and imported into Geomagic Wrap 12.0 for further processing. Due to the poor surface smoothness of the 3D model constructed by Mimics 21.0, it is prone to stress concentration problems and cannot meet experimental requirements. Therefore, it is necessary to optimize the model and perform surface fitting [21, 22]. Taking L2 vertebrae as an example, the optimization method of the model is as follows. First, use polygon commands to redraw the mesh, set the target edge length to 0.7 mm, apply the mesh and delete the boundaries. Then, use transparent commands to inspect and remove impurities inside the vertebrae and repair the holes. Next, the nail like structures on the surface of the mid vertebrae are feature removed, followed by detail polishing and smoothing. Finally, use the grid doctor command to check and automatically repair model issues to obtain a smooth L2 vertebral model. After optimization, although the surface smoothness of the model has significantly increased, the internal bone has not been effectively treated. Therefore, it is necessary to construct cancellous bone inside the vertebrae. The steps for constructing cancellous bone are as follows. First, the optimized vertebral model is copied and its posterior structure is deleted, retaining only the anterior vertebral body. Then, by offsetting the vertebral body inward, the corresponding cancellous bone model can be obtained, with a vertebral body offset distance of 1 mm. After constructing the trabecular bone model, it can be subjected to surface fitting processing. The surface fitting method is as follows. First, reduce the sensitivity to curvature, manually draw and adjust the vertebral contour lines, and remove duplicate contour lines to ensure accurate drawing of special stress areas. Then, set a reasonable number of surface patches, analyze and manually adjust the intersecting paths, and generate more regular four sided patches. Next, adjust the error grille until the error prompt is eliminated. Finally, fit the NURBS surface. The 3D FEA model of L2 vertebrae is displayed in Figure 2.

In Figure 2, after surface optimization, a three‐dimensional solid model of the L2 vertebrae can be obtained. Following the above steps, vertebral body models and trabecular bone models of other vertebrae are generated, and all models are exported in STP format for subsequent model combination. Each vertebral body model and cancellous bone model are imported into Solidworks 2019 for assembly, and cortical bone models are constructed. Taking L1 vertebrae as an example, the construction method of cortical bone model is as follows. Hide all models except for the L1 vertebral model and cancellous bone model, and copy the cancellous bone model of the L1 vertebra. Next, hide the original L1 trabecular bone model and click on the cortical bone model and the copied L1 trabecular bone model. Then, delete the copied L1 trabecular bone model to obtain the cortical bone model of L1. After constructing the cortical bone model, the lumbar disc model can be constructed. Taking the L1−2 lumbar disc as an example, its construction method is as follows. First, hide the bone model except for L1 and L2. Then, create a reference plane on the lower surface of L1 and draw a circular outline. Next, a preliminary model of the lumbar intervertebral disc was formed by stretching the feature stretching protrusion downwards by 30 mm. Use the equidistant surface tool to generate surfaces 1 and 2, and divide and remove both sides of the convex surface. Generate surfaces 3 and 4 by equidistant 0.5 mm from the surface of the preliminary model, forming the endplate of L1−2. The endplate and surface are hidden, and the nucleus pulposus contour is drawn on the reference plane, while the convex platform is segmented to obtain the nucleus pulposus model. Then, the contour line of the fiber ring is drawn in the above model, and the convex platform is segmented again to obtain a three‐layer fiber ring. The L1−2 lumbar disc model can be obtained through the above operation. Finally, the above model is saved in X_T format and assembled into the lumbar spine model. After assembling the model, it is imported into Abaqus 2021 for assignment. The material properties of the lumbar spine model are displayed in Table 3.

**Table 3:** Material properties of the lumbar spine model.

Region	Modulus of elasticity (MPa)	Poisson ratio	Type of constitutive model
Cortical bone	12,000	0.3	Linear elastic constitutive model
Outer layer of fibrous ring	550	0.3	Horizontally isotropic linear elasticity
Fiber ring second layer	490	0.3	Horizontally isotropic linear elasticity
Fiber ring third layer	440	0.3	Horizontally isotropic linear elasticity
Cancellous bone	100	0.2	Hyperelastic constitutive model
Cartilaginous endplate	25	0.3	Linear elastic constitutive model
Ligamentum flavum	15	0.3	Linear elastic constitutive model
Posterior longitudinal ligament	10	0.3	Linear elastic constitutive model
Interspinous ligament	10	0.3	Linear elastic constitutive model
Intertransvers ligament	10	0.3	Linear elastic constitutive model
Supraspinal ligament	8	0.3	Linear elastic constitutive model
Anterior longitudinal	8	0.3	Linear elastic constitutive model
Nucleus pulposus	1	0.5	Linear elastic constitutive model

According to Table 3, the elastic moduli of cortical bone, outer layer of fibrous ring, fiber ring second layer, fiber ring third layer, and cancellous bone are 12,000, 550, 490, 440, and 100 MPa, with Poisson’s ratios of 0.3, 0.3, 0.3, 0.3, and 0.2, respectively. After assigning values to the materials, the mesh of the model can be divided. Considering the computational accuracy of the model, the selected cell size for the study is 2 mm and its shape is tetrahedral. It is worth noting that, in order to simplify the study, nonlinear behavior (such as material yield and large deformation) and time‐dependent characteristics (such as viscoelasticity) were not considered. Figure 3 displays the FEA model and mesh division.

Figure 3FEA model and mesh division of lumbar spine. (a) Mesh division. (b) Finite element model of lumbar spine.

**Figure:**
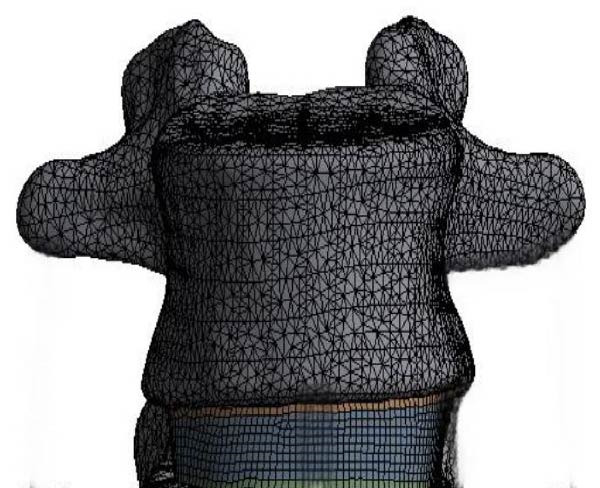
(a)

**Figure:**
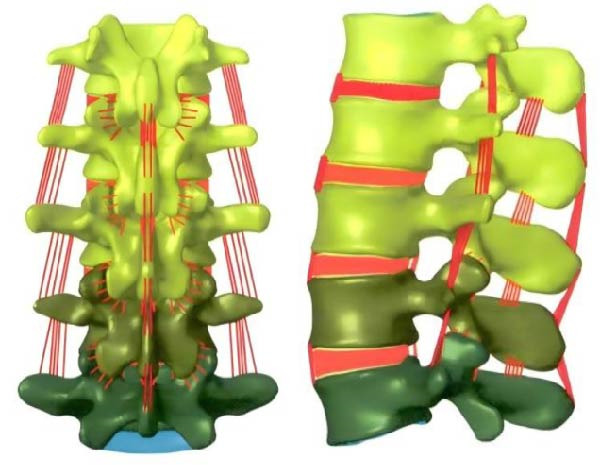
(b)

According to Figure 3, the FEA model of the lumbar spine includes five pieces of cancellous bone and five pieces of cortical bone, four pieces of nucleus pulposus and eight pieces of endplate, and 12 pieces of annulus fibrosus and six pieces of ligaments. The number of cells in the entire model is 790,783, and the node is 183,995. The boundary conditions of the model are determined based on the flexion angle, peak moment, and vertical load of the lumbar spine. Specifically, fixed constraints are applied at the bottom of the model, namely the L5 vertebral endplate, to simulate the connection between the lumbar spine and the pelvis. At the top of the model, namely the endplate of the L1 vertebral body, a vertical compressive load is applied, which is evenly distributed at various nodes on the surface, simulating the effect of body weight on the lumbar spine. The upper endplate of the L1 is subjected to forward flexion, backward extension, lateral flexion, and rotational torque loads to simulate the stress situation of the lumbar spine during physiological movement. The contact relationship between each vertebral body in the model is defined as face‐to‐face binding contact, and the contact behavior is set as antisymmetric constraint to simulate the interaction between vertebral bodies. The dynamic analysis of lumbar spine torque is based on the inverse dynamics modeling framework, and the algorithm used is the Newton Euler recursive algorithm. This algorithm is based on the inverse logic of “kinematic data → joint torque,” which uses known motion results (human segment position, velocity, acceleration) and external loads (ground reaction force, barbell gravity) to infer the dynamic torque of the lumbar spine and other joints. The core formula follows the rigid body dynamic equilibrium Equation (1).

(1) τ=Iα+ωIω+mrc×ag−Fext×rc.

In Equation (1), *τ* represents joint torque, *I* represents segment moment of inertia, *α* is the angular acceleration, *ω* is the angular velocity, *m* is the quality of the segment, *r*_*c*_ is the centroid position vector, *a*_*g*_ is the acceleration due to gravity;, and *F*_ext_ is an external force.

### 2.3. Data Processing and Statistical Methods

Data processing method: In order to improve data quality, a fourth‐order Butterworth filter is used to filter the data. When filtering, the cutoff frequencies for kinematics and dynamics are 10 and 100 Hz, respectively. When conducting theoretical calculations, the human torso is regarded as a regular elliptical frustum. By calculating the regular elliptical frustum and combining it with the basic parameters of the human joints, the mass of the torso above the L1 vertebral body is calculated.

Data statistics method: SPSS25.0 and Excel are used to analyze the data. All parameters are represented in the form of X ± Y.

## 3. Results

### 3.1. Kinematic Characteristic Analysis of Lumbar Spine Under Different Hard Pull Actions

To investigate the kinematic characteristics of the lumbar spine under different hard pull actions, FEA is conducted. When conducting FEA, a load of 1425N needs to be applied to the upper surface of the L1 vertebra due to the 120 kg hard pull performed by the volunteers. The flexion angle and torque changes of various hard pulling methods are shown in Figure 4.

Figure 4Fleling angle and torque changes of the lumbar spine. (a) Flexion angle of lumbar spine. (b) The torque of the lumbar spine. DL, deadlift; SLDL, stiff‐legged deadlift; TBDL, trap bar deadlift.

**Figure:**
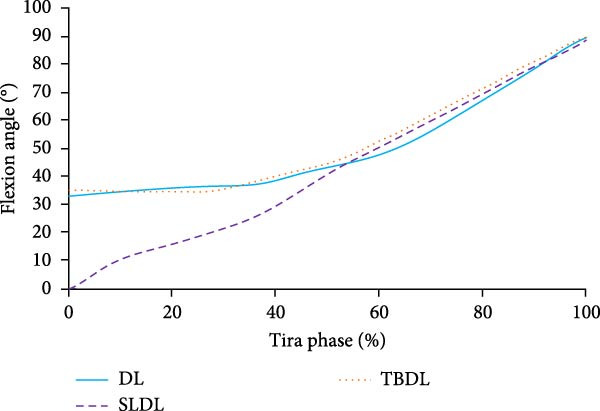
(a)

**Figure:**
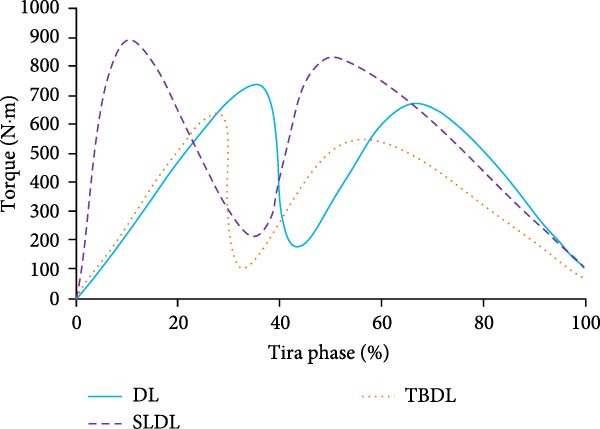
(b)

From Figure 4a, the lumbar spine flexion angle changes in traditional hard pull and hexagonal barbell hard pull was basically the same, and the amplitude was relatively small, with flexion angle ranges of 32°–90° and 35°–90°, respectively. The range of flexion angle changes for the lumbar spine with straight leg hard pull was relatively large, ranging from 0° to 90°. From Figure 4b, under different hard pulling actions, there were two peaks in the torque of the lumbar spine, and the second peak was smaller than the first peak. The peak torque of the straight leg hard pull was much greater than that of other hard pull actions, and it appeared earlier. The first peak torques for traditional hard pull, straight leg hard pull, and hexagonal barbell hard pull were 749, 893, and 640 N∙m, respectively. The stress distribution of the lumbar spine hard pulling actions is displayed in Figure 5.

Figure 5Stress distribution of the lumbar spine. (a) DL, (b) SLDL, and (c) TBDL.

**Figure:**
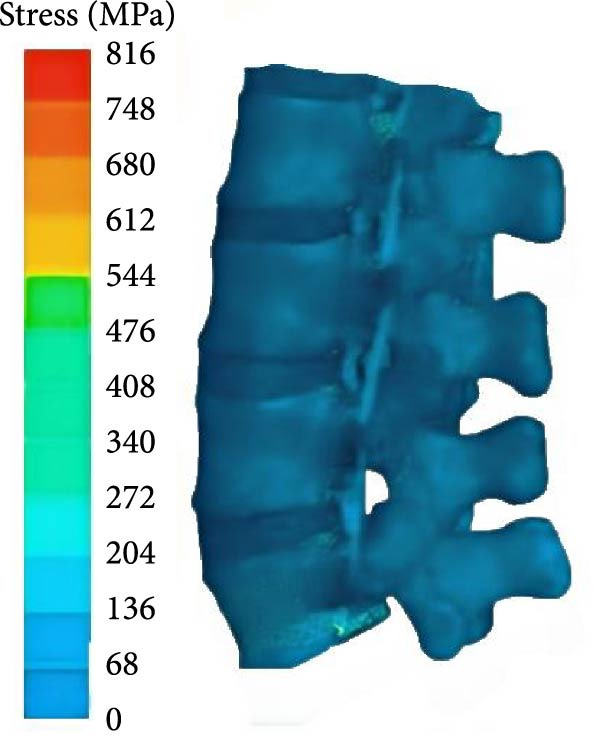
(a)

**Figure:**
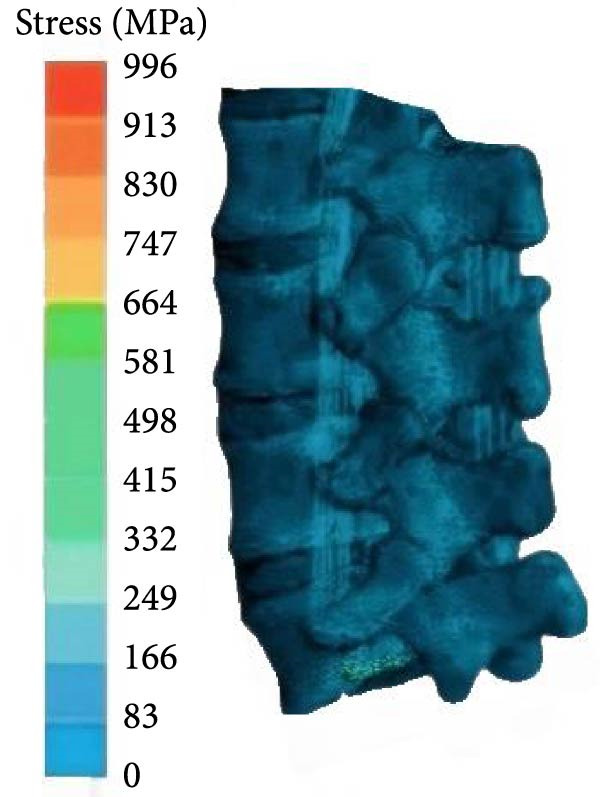
(b)

**Figure:**
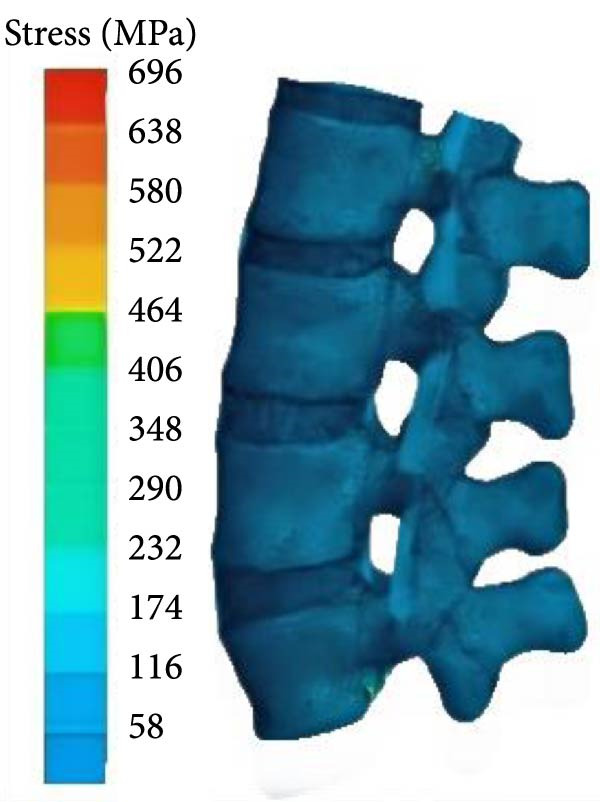
(c)

From Figure 5, under various hard pulling actions, the maximum stress of the lumbar spine appeared at the posterior lower edge of the L5 vertebral body, and the stress of straight leg hard pull was the highest, followed by traditional hard pulling. The maximum stresses for traditional hard pull, straight leg hard pull, and hexagonal barbell hard pull were 816, 996, and 696 MPa, respectively.

### 3.2. Stress Distribution Characteristics of Lumbar Joints Under Different Hard Pulling Actions

**Figure 6:**
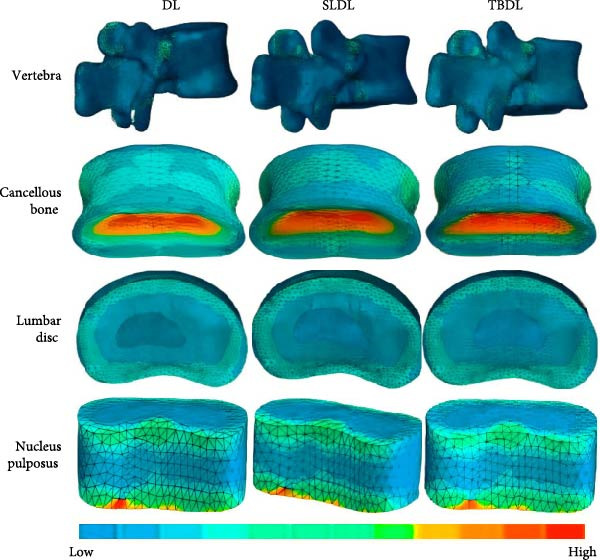
Stress distribution of vertebral L1 and the corresponding lumbar disc and nucleus pulposus.

To further analyze the stress distribution of the lumbar spine under various hard pull actions, the study analyzed different vertebral joints. The stress distribution of the vertebral body, corresponding lumbar intervertebral disc, and nucleus pulposus is shown in Figure 6.

In Figure 6, the vertebral body L1, corresponding lumbar intervertebral disc, and nucleus pulposus were basically consistent under different hard pulling actions. Stress concentration mainly occurred at the pedicle, transverse process, and posterior upper/lower edge of the vertebral body. Compared to traditional hard pull and hexagonal barbell hard pull, straight leg hard pull required greater stress. For cancellous bone, stress concentration under different hard pulling actions mainly occurred in the middle position. For the L1−2 lumbar disc, the area was mainly located at the peripheral edges. For the L1−2 nucleus pulposus, stress concentration mainly occurred at the upper and lower edges. For the L2 vertebral body, the location was consistent with that of the L1 vertebral body. For cancellous bone, stress concentration was equally important in the central region. For the L2−3 lumbar disc, the area was also located at the edges of the periphery, but compared to L1−2, the stress concentration area was expanded. For the L2−3 nucleus pulposus, the stress was mainly concentrated at the lower edge position. The stress concentration of L3 vertebral body was basically consistent with that of L1 and L2. For cancellous bone, although the stress concentration area was still in the middle of the nucleus pulposus, its range was significantly expanded. For the L3−4 lumbar disc, the area was also located at the edges of the periphery, but compared to L2−3, the stress concentration area was slightly reduced. For the L3−4 nucleus pulposus, the stress concentration area significantly expanded and was mainly distributed to the outer side of the nucleus pulposus. The stress distribution of vertebral body L4 was consistent with that of L1−L3. For cancellous bone, its stress distribution was basically consistent with L3. For the L4−5 lumbar disc, the area was located at the anterior and posterior edges. For the L4−5 nucleus pulposus, the area was located at the lower edge. The stress distribution of vertebral body L5 was basically consistent with that of L1–L4. However, compared to other trabecular bones, the stress concentration in L5 trabecular bone was more severe, with stress concentration areas almost covering the entire trabecular bone. Due to the high risk of injury to the L4−5 lumbar disc, a quantitative difference analysis is conducted in the study. The quantitative difference analysis of the hard pull of the hexagonal barbell is displayed in Table 4.

**Table 4:** Results of quantitative difference analysis of hexagonal barbell pull.

Region	Von‐Mises (MPa)		Coefficient of variation	Quantitative differences	
	Mean stress	Standard deviation		Mean stress	Standard deviation
A	0.60	0.19	0.33	1.63	1.22
B	0.65	0.23	0.41	1.75	1.63
C	0.76	0.41	0.58	2.11	2.47
D	0.77	0.30	0.39	2.13	1.61
E	0.28	0.05	0.18	0.00	0.00
F	0.99	0.42	0.46	2.69	2.03
G	1.01	0.43	0.43	2.72	1.74
H	1.16	0.61	0.55	3.03	2.39
I	1.15	1.02	0.97	3.03	3.50

*Note:* A and C are the upper left and right corners, B and H are the upper and lower middle regions, D and F are the left and right middle regions, C and I are the lower left and right corners, and E is the central region of the lumbar disc.

**Figure 7:**
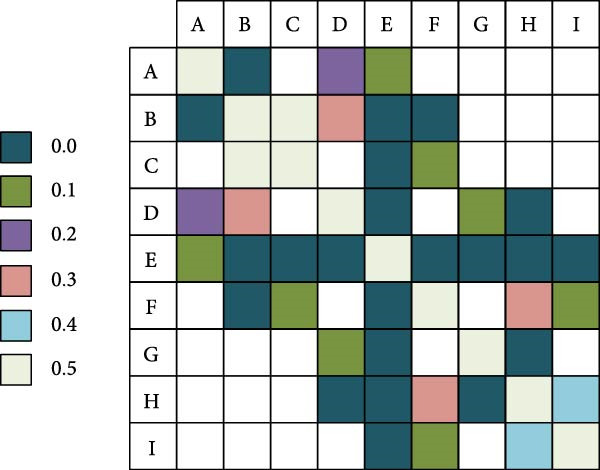
Significant differences in various regions of lumbar disc L4−5.

In Table 4, the significant difference existed in Von‐Mises stress between Region A and Region E, with a quantitative difference value of 1.22. This value was greater than the *β* of 0.8 but less than the *γ* of 1.27. The difference between the two regions was significant but did not reach a very significant level. In contrast, the Von‐Mises stress differences between other regions and Region E were more significant, with quantitative differences exceeding the γ of 1.27. From other Von‐Mises stresses, the average Von‐Mises stress in Region E was only 0.28 MPa, which was lower than that in the surrounding area. When the hexagonal barbell was pulled down hard, the area of the L4−5 lumbar intervertebral disc was around the periphery. The significant differences in various regions of lumbar disc L4−5 are displayed in Figure 7.

In Figure 7, the Von‐Mises stress in the Region E where the nucleus pulposus was located showed significant differences compared to the surrounding eight regions. In addition, there were significant differences in Von‐Mises stress among B–F, D–H, and G–H regions, with very low *p*‐values (less than 0.01), which further emphasized the specificity of stress distribution in these regions. However, although the Von‐Mises stress difference between the A–B and C–F regions is significant, it has not reached the significance level compared to the Region E, with *p*‐values from 0.01 to 0.05. This indicates that although there are stress differences between these regions, they are not as strong as the differences with Region E. Finally, no obvious difference existed in Von‐Mises stress among A–D, B–C, B–D, D–G, F–H, F–I, and H–I regions. The quantitative difference analysis results of straight leg hard pull are displayed in Table 5.

**Table 5:** Results of quantitative difference analysis of the straight leg hard pull.

Region	Von‐Mises (MPa)		Coefficient of variation	Quantitative differences	
	Mean stress	Standard deviation		Mean stress	Standard deviation
A	1.13	0.28	0.32	2.38	1.31
B	1.28	0.39	0.37	2.52	1.63
C	1.51	0.51	0.61	2.64	2.61
D	1.22	0.30	0.42	2.83	1.78
E	1.15	0.16	0.25	0.00	0.00
F	1.93	0.47	0.53	3.12	2.11
G	1.29	0.51	0.62	3.36	1.96
H	1.45	0.61	0.71	4.15	2.55
I	1.57	0.96	0.96	4.13	3.67

According to Table 5, the significant differences existed in Von‐Mises stress between other regions and Region E, and their quantitative differences exceeded the γ of 1.27. From other Von‐Mises stresses, the average Von‐Mises stress in Region E was only 1.15 MPa, which was lower than that in the surrounding area. During the straight leg hard pull, the stress concentration area of the L4−5 lumbar disc is still in the surrounding position. The quantitative difference analysis results of traditional hard pull are displayed in Table 6.

**Table 6:** Results of quantitative difference analysis with conventional hard pull.

Region	Von‐Mises (MPa)		Coefficient of variation	Quantitative differences	
	Mean stress	Standard deviation		Mean stress	Standard deviation
A	0.97	0.21	0.35	1.72	1.24
B	1.06	0.41	0.39	1.96	1.67
C	1.33	0.39	0.65	2.32	1.76
D	1.13	0.47	0.47	2.45	1.85
E	0.82	0.22	0.29	0.00	0.00
F	1.86	0.59	0.58	2.78	1.94
G	1.11	0.61	0.64	2.92	1.82
H	1.38	053	0.69	3.43	2.15
I	1.41	0.88	0.99	3.43	2.26

In Table 6, the significant difference existed in Von‐Mises stress between Region A and Region E, with a quantitative difference value of 1.24. This value was greater than the *β* of 0.8 but less than the γ of 1.27. The difference between the two regions is significant but has not reached a very significant level. There were significant differences between other regions and Region E, and their quantitative differences exceeded the γ of 1.27. From other Von‐Mises stresses, the average Von‐Mises stress in Region E was only 0.82 MPa, which was lower than that in the surrounding area. Under traditional hard pulling, the stress concentration area of the L4−5 lumbar disc is still in the surrounding positions. The peak stress changes of each vertebral body and trabecular bone are shown in Figure 8.

Figure 8Changes in peak stress of each vertebral body and trabecular bone. (a) Peak stress of vertebral body, (b) peak stress of cancellous bone.

**Figure:**
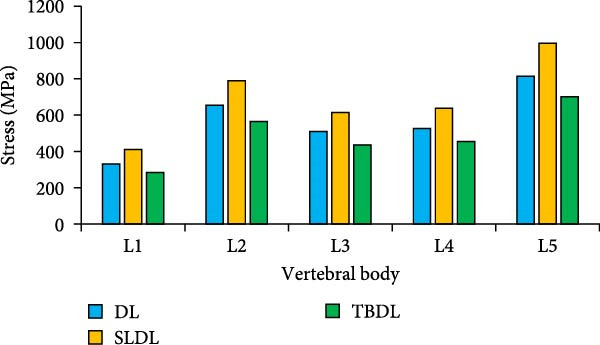
(a)

**Figure:**
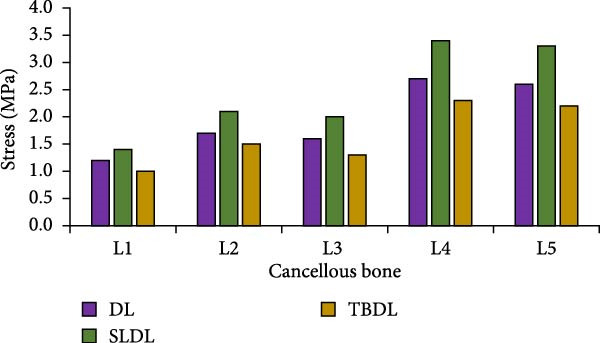
(b)

As shown in Figure 8a, compared to traditional hard pulling and hexagonal barbell hard pulling, the peak stress of straight leg hard pull was larger, at least 20% higher. Among all vertebral bodies, the peak stress borne by L5 vertebral body was much greater than that of other vertebral bodies. The peak stress values of L5 vertebrae in traditional hard pull, straight leg hard pull, and hexagonal barbell hard pull were 815, 997, and 701 MPa, respectively. As shown in Figure 8b, for cancellous bone, the peak stress of straight leg hard pull was higher than other hard pulling actions, increasing by at least 16%. And among all the cancellous bones, the peak stress of L5 trabecular bone was much higher than that of other trabecular bones. Under different hard pulling actions, the peak stress values of L5 trabecular bone were 2.6, 3.3, and 2.2 MPa, respectively. The above results indicate that for weightlifters, the L5 vertebral body bears greater pressure. The peak stress changes of each lumbar intervertebral disc and nucleus pulposus are shown in Figure 9.

Figure 9Peak stress values of lumbar intervertebral discs and nucleus pulposus. (a) Peak stress of lumbar intervertebral disc, (b) peak stress of nucleus pulposus.

**Figure:**
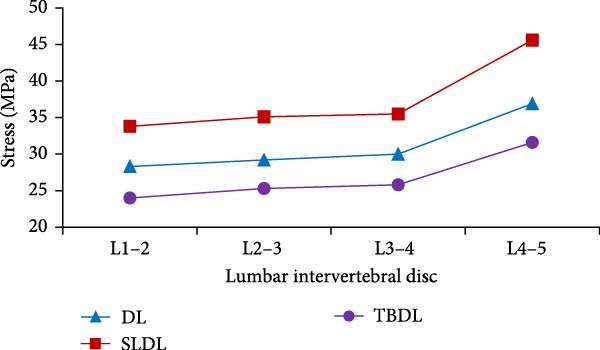
(a)

**Figure:**
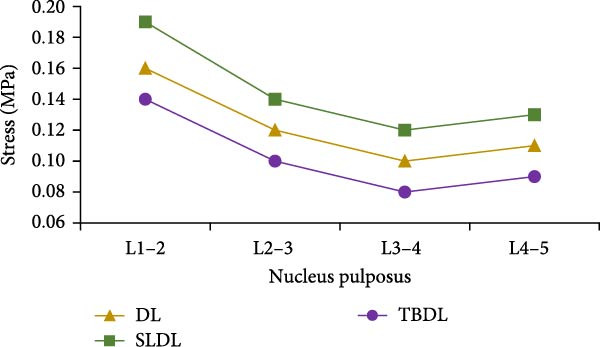
(b)

According to Figure 9a, among various hard pulling actions, the peak stress of the lumbar intervertebral disc during straight leg hard pull was the highest, which was at least 18% higher than other actions. For each lumbar disc, the peak stress gradually increased from top to bottom. Under traditional hard pull, straight leg hard pull, and hexagonal barbell hard pull actions, the peak stress of L4−5 lumbar intervertebral disc was the highest, at 36.9, 45.6, and 31.6 MPa, respectively. From Figure 9b, compared to other actions, the peak stress of the nucleus pulposus was greater when the straight leg was pulled hard. As for each nucleus pulposus, its peak stress first decreased and then increased from top to bottom. Under different actions, the peak stress of L3−4 nucleus pulposus was the smallest, at 0.10, 0.12, and 0.08 MPa, respectively. In order to verify the effectiveness of the finite element model, a comparative verification was conducted using authoritative literature in the field of lumbar spine biomechanics.

According to Table 7, the relative error between the stress and stiffness indicators of the FEA model and the data from three authoritative literature is ≤4%, and all simulated values fall within the physiological range reported in the literature (such as intervertebral disc stress 35–40 MPa, trabecular bone stress 2.0–3.0 MPa), indicating that the mechanical response of the model conforms to the physiological characteristics of the human lumbar spine and the stress distribution analysis results are reliable. The sensitivity analysis results of the article parameters are shown in Table 8.

**Table 7:** Verification results of finite element model.

Validation indicators	Peak stress of L5 vertebral cortical bone (MPa)	Peak stress of L4–L5 intervertebral disc (MPa)	Peak stress of L5 trabecular bone (MPa)	Lumbar spine overall compression stiffness (N/mm)
FEA simulation values	815	36.9	2.6	1250
Literature data (source)	820 ± 30 (Ke et al. 2021) [8]	37.5 ± 2.0 (Zhao et al. 2023) [10]	2.5 ± 0.3 (Spina et al. 2021) [9]	1230 ± 50 (Ke et al. 2021) [8]
Relative error	0.6%	1.6%	4.0%	1.6%
Conformance statement	The stress values are highly consistent	The range of intervertebral disc stress that conforms to literature reports	Within the discrete range of literature data	The stress values are highly consistent

**Table 8:** Results of parameter sensitivity analysis.

Key parameters	Parameter fluctuation amplitude (%)	Peak stress change rate of L5 vertebral cortical bone	Sensitivity level
Elastic modulus of cortical bone	±10	[−8.2%, 8.5%]	High sensitive
Elastic modulus of nucleus pulposus	±20	[−2.3%, 2.1%]	Low sensitivity
Vertical static load	±5	[4.6%, 4.8%]	Medium sensitivity
Dynamic peak torque	±5	[−4.7%, 4.9%]	Medium sensitivity

According to Table 8, the elastic modulus of vertebral cortical bone is the only highly sensitive parameter, but the original text was accurately calibrated using HU values from CT images (ensuring that the elastic modulus is within the range of 12,000 ± 10%), and the physiological fluctuations of this parameter have been included in the simulation error, making the results reliable. The impact of fluctuations in the elastic modulus of the nucleus pulposus (low sensitivity) and vertical load/dynamic torque (moderate sensitivity) on vertebral stress is ≤5%, which is much lower than the stress difference between “straight leg hard pull vs hexagonal hard pull” (997 vs 701 MPa, difference of 30%). This indicates that even with slight fluctuations in parameters, the core conclusion of the “damage risk gradient of the three hard pull actions” remains unchanged, and the model has good stability.

## 4. Discussion

Weightlifting can be traced back to the production and labor practices of ancient humans. In ancient Greece and Rome, weightlifting was used as a way to assess the strength of warriors. In China, weightlifting activities have been recorded as early as the Chu and Han dynasties over 2000 years ago, such as wielding large swords, stone poles, stone locks, etc. Modern weightlifting originated in 18th century Europe. At that time, circus troupes in London, England often had weightlifting performances. In the early 19th century, weightlifting clubs were established in England. At the first Olympic Games in 1896, weightlifting was taken as an official competition event [23]. With the passage of time, weightlifting gradually developed into the well‐known form today, which includes two events: snatch and clean‐and‐jerk. Although weightlifting is a beneficial exercise that enhances strength and muscles, it also comes with certain risks. Weightlifters have a very high probability of waist injury during long‐term training due to the high‐intensity and overloaded exercise on their waist. According to a survey, the waist injury rate of weightlifters is over 95.2%, with the proportion of lumbar and sacral spine muscle injuries accounting for 77.5% [24, 25]. The lumbar spine is highly susceptible to injury during weightlifting. Therefore, to explore the kinematic characteristics of the lumbar spine in weightlifting, FEA is conducted on the lumbar spine under different hard pull actions.

The research results showed that the changes in lumbar flexion angle between traditional hard pull and hexagonal barbell hard pull were basically the same, and the amplitude of changes was relatively small, with flexion angle ranges of 58° and 55°, respectively. The range of flexion angle changes for the lumbar spine with straight leg hard pull was relatively large, at 90°. This is because compared to other hard pull actions, the initial angle of the lumbar spine during straight leg hard pull is the smallest, almost parallel to the ground. At the same time, under different hard pulling actions, there were two peaks in the torque of the lumbar spine, and the second peak was smaller than the first peak. The peak torque of the straight leg hard pull was much greater than that of other hard pull actions, and it appeared earlier. The first peak moments for traditional hard pull, straight leg hard pull, and hexagonal barbell hard pull were 749, 893, and 640 N∙m, respectively. For each lumbar joint, the stress distribution of the lumbar vertebrae L1, L2, L3, L4, and L5, as well as the corresponding lumbar intervertebral discs and nucleus pulposus, was basically the same under different hard pulling actions. In all hard pull actions, the peak stress borne by the L5 vertebral body was much greater than that of other vertebral bodies, and the peak stress of the L5 trabecular bone was also much greater than that of other trabecular bones. Erdağı and Poyraz [26] found that weightlifters had a larger cross‐sectional area of the lumbar multifidus muscle at the L4–L5 vertebral level compared to sedentary individuals. In addition, compared to traditional hard pulling and hexagonal barbell hard pulling, the stress of straight leg hard pull was greater. Especially in the L5 vertebral body and L5 trabecular bone, the peak stress increased by at least 20%. The peak stress values of L5 vertebral body in traditional hard pull, straight leg hard pull, and hexagonal barbell hard pull were 815, 997, and 701 MPa, respectively, while the peak stress values of L5 trabecular bone were 2.6, 3.3, and 2.2 MPa, respectively.

In terms of stress concentration, vertebral stress concentration mainly occurred at the pedicle, transverse process, and posterior upper/lower edge of the vertebral body. For cancellous bone, stress concentration under different hard pulling actions mainly occurred in the middle position. As the vertebral body moved from L1 to L5, the range of stress concentration areas gradually expanded, especially in L5 trabecular bone, where the stress concentration area almost covered the entire trabecular bone. Comparing the stress of vertebral bodies and cancellous bone, the peak stress of vertebral bodies was much greater than that of cancellous bone. This is because the density of cancellous bone is relatively low, so it bears less stress [27, 28]. For vertebral bodies, they mainly relied on denser cortical bone to withstand pressure. For lumbar intervertebral discs, the stress concentration area appeared at the peripheral edges, and the stress concentration area increased from L1−2 to L4−5. For the nucleus pulposus, the stress concentration area was mainly located at the edge. This is because the nucleus pulposus has plasticity. When subjected to external forces, it changes shape and disperses stress to the annulus fibrosus [29, 30].

Based on the risk gradient of lumbar spine injury for three types of hard pull movements (straight leg hard pull > traditional hard pull > hexagonal barbell hard pull), this study combines training scenarios and population characteristics to develop selection criteria to avoid injuries caused by “blind use of high‐risk movements.” For novice/teenage athletes: prioritize hexagonal barbell hard pull. The original data shows that the peak stress of the L5 vertebral body (701 MPa), the stress of the L5 trabecular bone (2.2 MPa), and the stress of the L4−L5 intervertebral disc (31.6 MPa) are all below the corresponding injury/tolerance threshold, and the lumbar flexion angle (55°) is the smallest—this movement has the lightest mechanical burden on the lumbar spine and can be used as an introductory movement for basic strength training, reducing the risk of injury for beginners due to technical unfamiliarity and high stress. For athletes with a history of lumbar discomfort: straight leg hard pull is prohibited and traditional hard pull is limited. The L5 vertebral body stress (997 MPa) of straight leg hard pull is close to the cortical bone injury threshold (1000 MPa), and the L4–L5 intervertebral disc stress (45.6 MPa) exceeds the physiological tolerance threshold (40 MPa) by 14%, which can easily induce the recurrence of old injuries; If you need to improve the strength of the posterior muscles (such as the hamstring and gluteus maximus muscles), you can use “traditional hard pull+knee angle adjustment” (the knee angle is increased from the “slight flexion” of traditional hard pull to 15°–20°). According to the original data, this adjustment can reduce the peak torque of the lumbar spine by about 8%–10% (from 749 to 680–699 N∙m), indirectly reducing the stress on the L5 vertebral body. Periodic training for high‐level athletes: incorporating three types of movements into different training stages. During the pre match strength enhancement stage (load of 80%–90% RM), traditional hard pulling can be used for a short period of time (frequency should be controlled to ≤2 times/week) to avoid straight leg hard pulling; The basic training phase during the off‐season mainly involves hexagonal barbell hard pulling (accounting for 60%–70% of the hard pulling training), which accumulates strength through low stress and reduces lumbar fatigue injury.

In summary, in weightlifting, due to the greater flexion angle changes and stress caused by straight leg stretching, the risk of lumbar spine injury is higher compared to other stretching methods. Meanwhile, due to the higher peak stress and more pronounced stress concentration of the L5 lumbar vertebrae compared to other vertebrae, the risk of injury during weightlifting is much higher than that of other lumbar vertebrae. In weightlifting, in order to protect the lumbar spine, it is advisable to avoid using straight leg stretching as much as possible, and certain protective measures should be taken for the L5 lumbar spine.

## 5. Conclusion

In summary, the research conducted experiments and FEA to explore the effects of various hard pull actions on the lumbar spine in weightlifting. The research results indicate that due to its larger lumbar flexion angles and higher peak stress, straight leg hard pull poses a significantly higher risk of lumbar injury than traditional hard pulling and hexagonal barbell hard pulling. The “excessive pressure” of straight leg hard pulling on the lumbar spine is reflected in: the cortical bone stress of L5 vertebral body is close to the damage threshold (997 vs 1000 MPa), the trabecular bone stress of L5 exceeds the fatigue threshold (3.3 vs 3.0 MPa), and the intervertebral disc stress of L4–L5 significantly exceeds the standard (45.6 vs 40 MPa). All three core structures are at high risk of damage. Especially in the L5 vertebral body and trabecular bone area, stress concentration caused by stiff leg pulling was more severe, which may be one of the main causes of lumbar spine injuries in weightlifters. At the same time, the study also found that under different hard pull actions, the torque change of the lumbar spine showed two peaks, among which the peak torque of the straight leg hard pull appeared earlier and had a larger value. This further confirms the potential threat of straight leg hard pull to the lumbar spine. The stress concentration areas of the lumbar disc and nucleus pulposus were mainly located at the edges, which were related to the plasticity of the nucleus pulposus. It can change shape and disperse stress to the annulus fibrosus when subjected to external forces. Based on the above research results, it is recommended that coaches and athletes should fully consider the impact of hard pull actions on the lumbar spine during weightlifting training and try to avoid or reduce the straight leg hard pull to reduce the lumbar spine injury. Meanwhile, the L5 vertebral body should be better protected, improving athletes’ physical control ability and lumbar stability through reasonable training plans and skill guidance. In addition, future research can further analyze the long‐term effects of various weightlifting movements on the lumbar spine, as well as how to effectively prevent and treat lumbar spine injuries through training and rehabilitation measures.

## Ethics Statement

This study was performed in line with the principles of the Declaration of Helsinki and approved by the Ethics Committee.

## Conflicts of Interest

The author declares no conflicts of interest.

## Author Contributions

Li Xiao made all the contributions related to this research.

## Funding

No funding was received for this research.

## Data Availability

The data that support the findings of this study are available from the corresponding author upon reasonable request.
